# Supplementary figures and images for: Prolonged β-adrenergic stimulation disperses ryanodine receptor clusters in cardiomyocytes and has implications for heart failure
Source: eLife. 2022 Aug 1;11:e77725. doi: 10.7554/eLife.77725 (PMC9410709; doi:10.7554/eLife.77725)

**Gel 1**

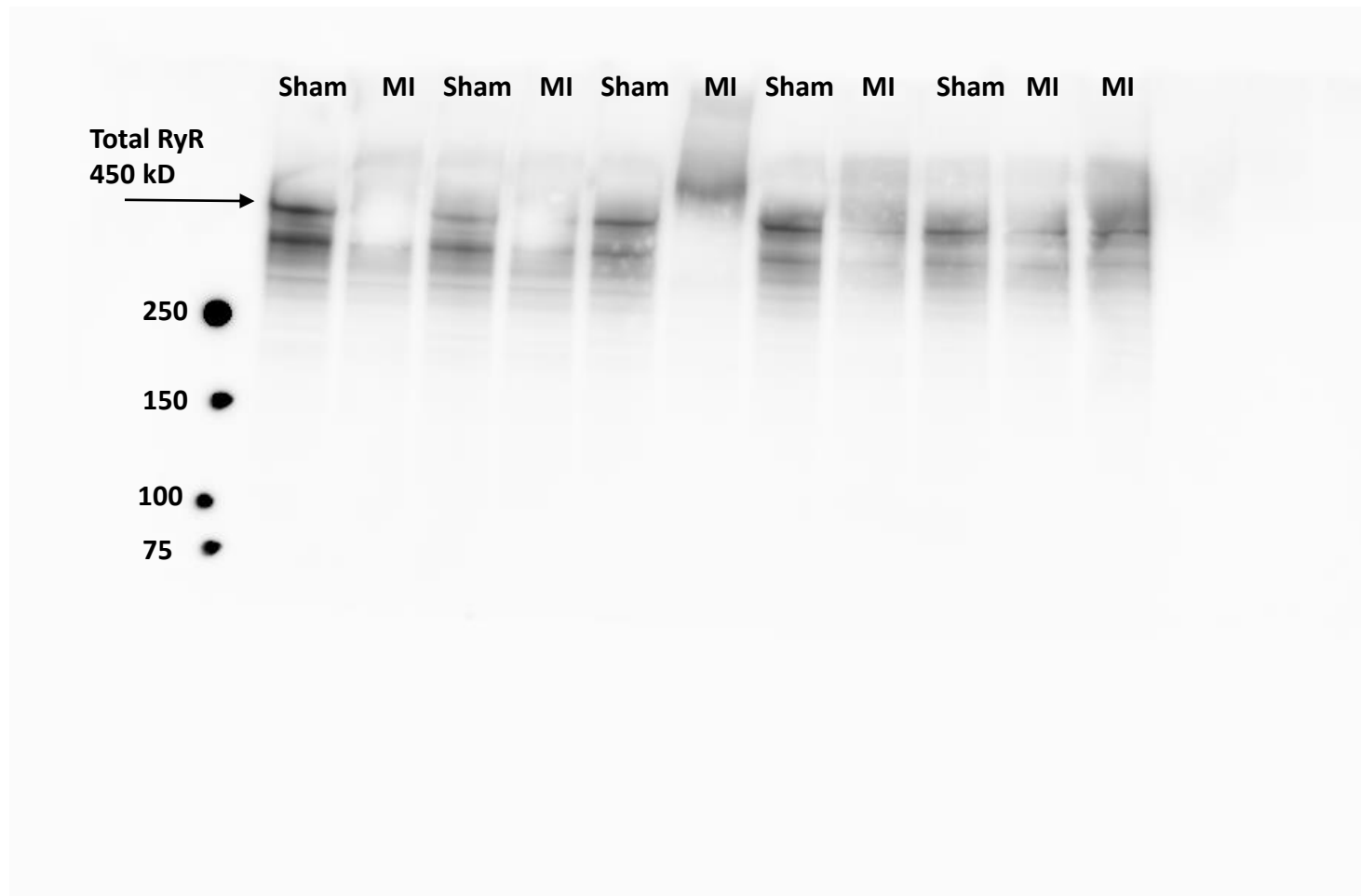

**Gel 2**

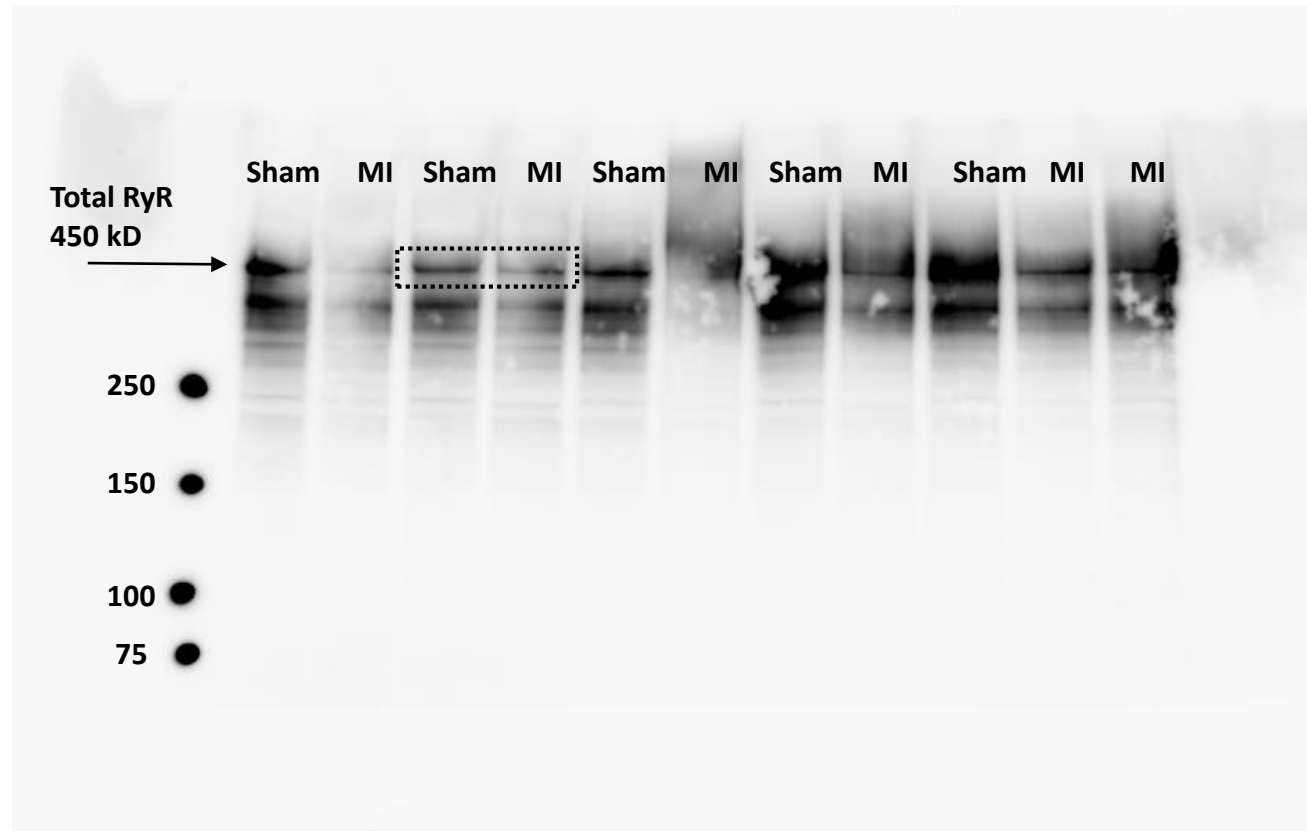

**Gel 1**

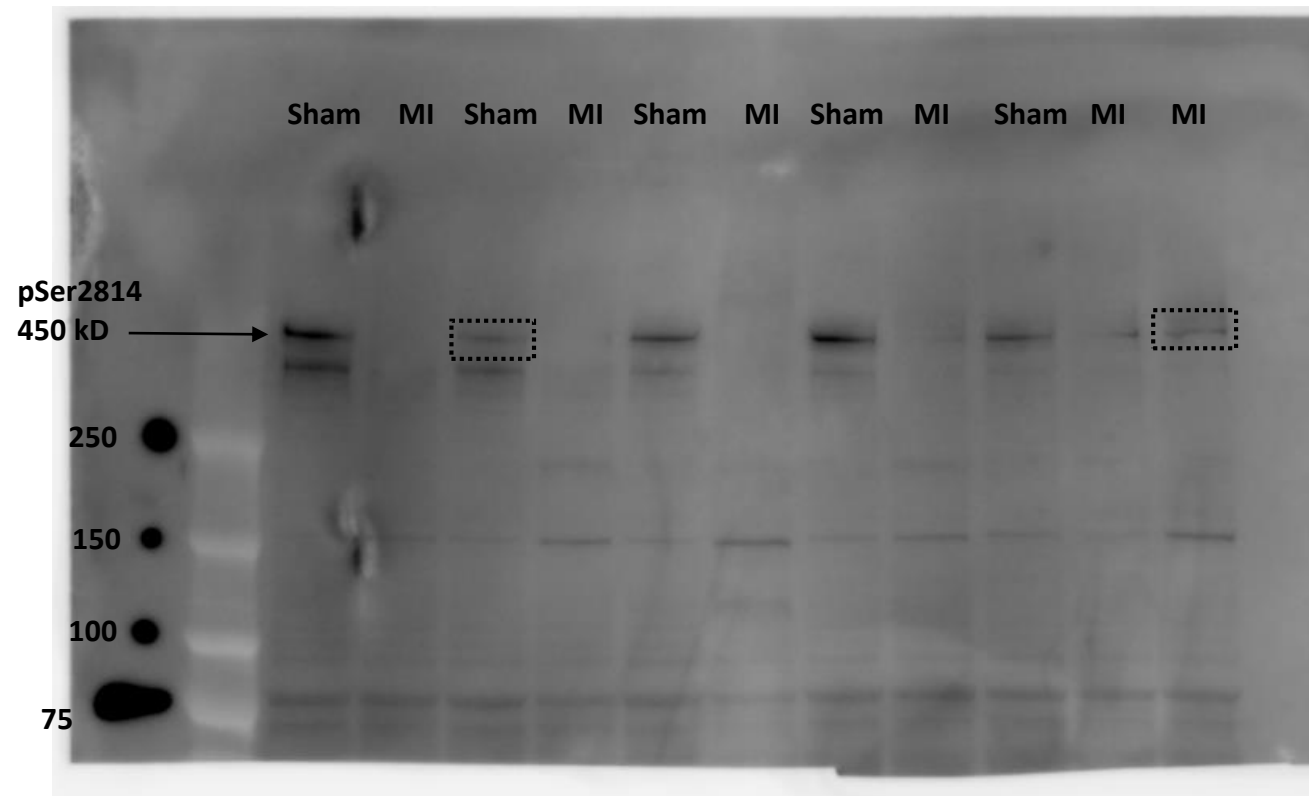

Gel 2

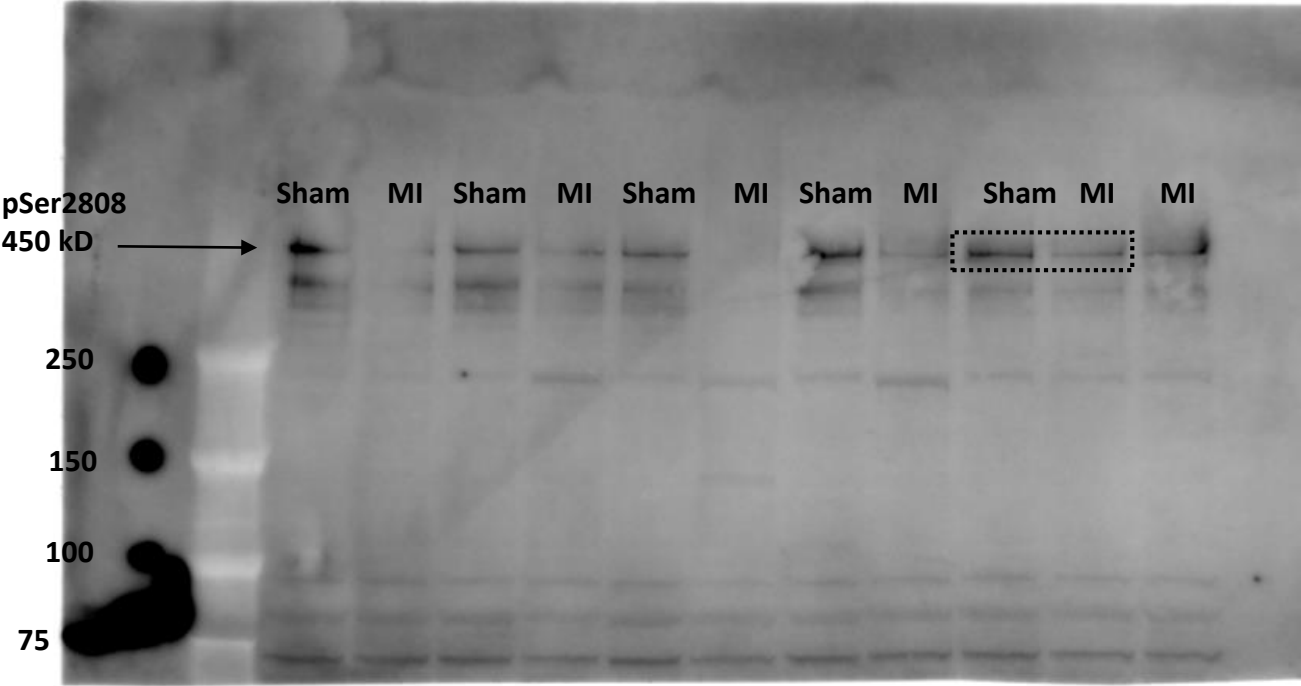

**Gel 1**

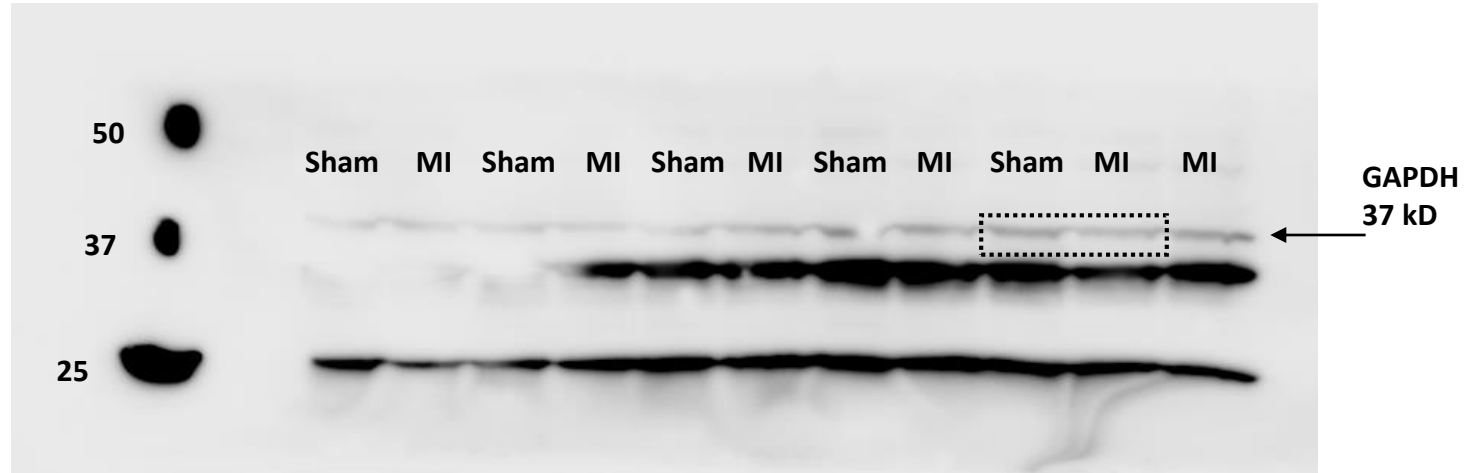

**Gel 2**

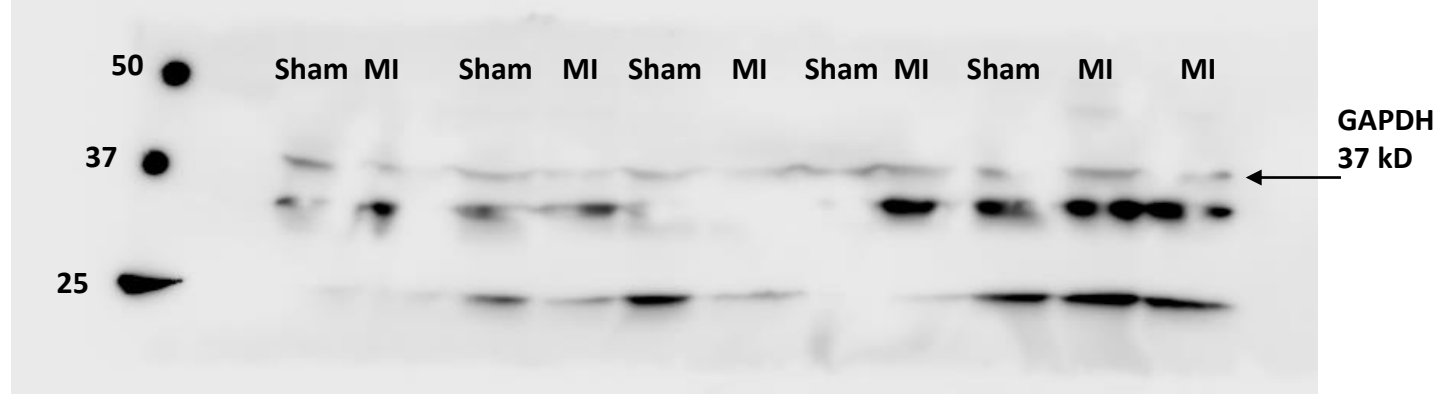

Supplement: Figure 6—source data 1. [file elife-77725-fig6-data1.zip › Source Data 1/Labelled Western blot of RyR2, 2814, 2808 and GAPDH.pdf]

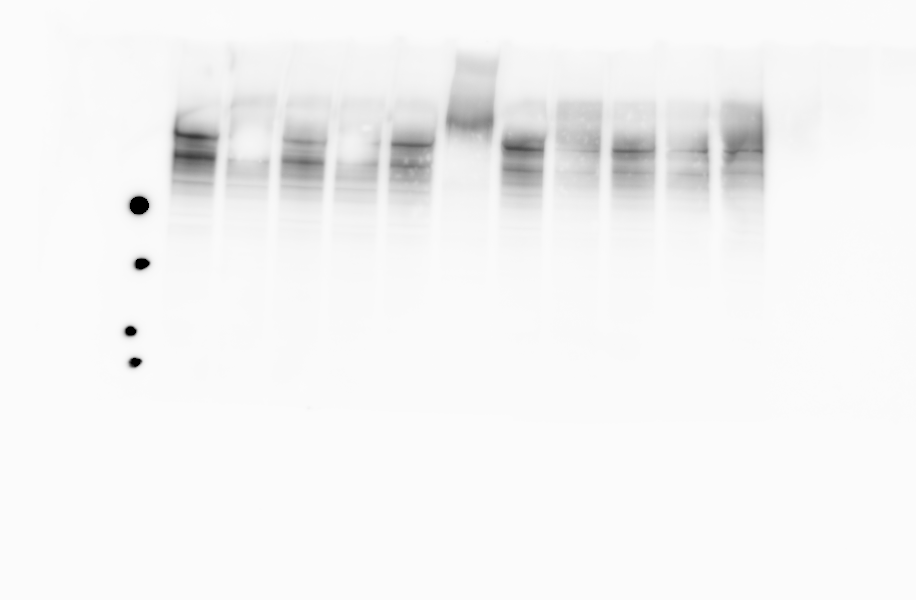

Supplement: Figure 6—source data 1. [file elife-77725-fig6-data1.zip › Source Data 1/Raw WB gel1 total RyR full.tif]

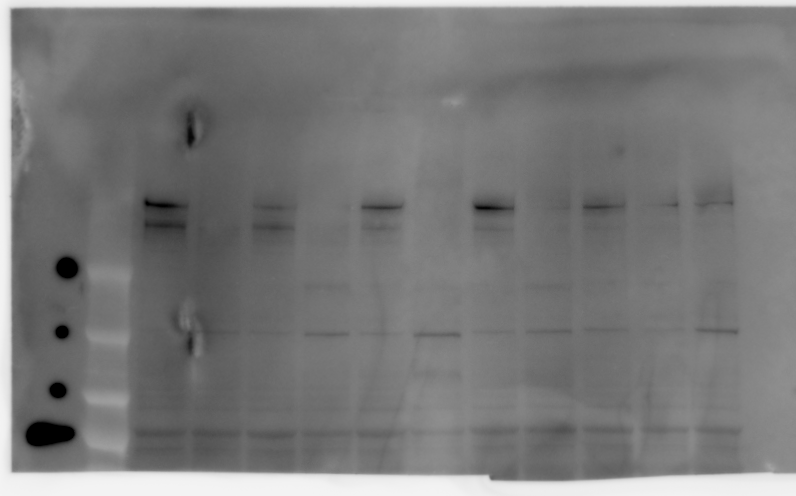

Supplement: Figure 6—source data 1. [file elife-77725-fig6-data1.zip › Source Data 1/Raw WB gel1a pser2814 full.tif]

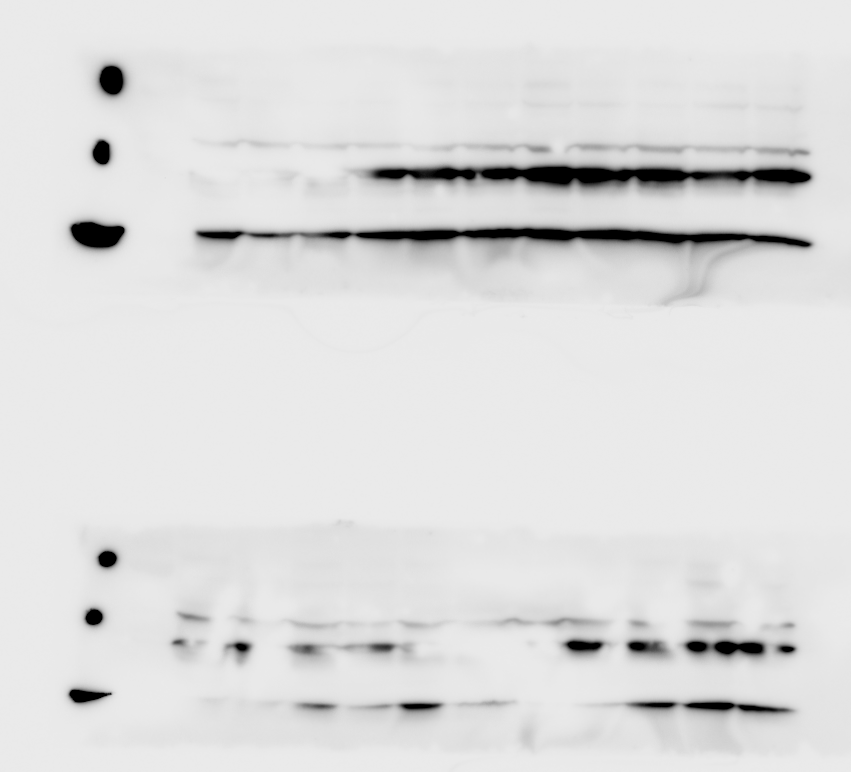

Supplement: Figure 6—source data 1. [file elife-77725-fig6-data1.zip › Source Data 1/Raw WB gel1b top gel2b bot gapdh.tif]

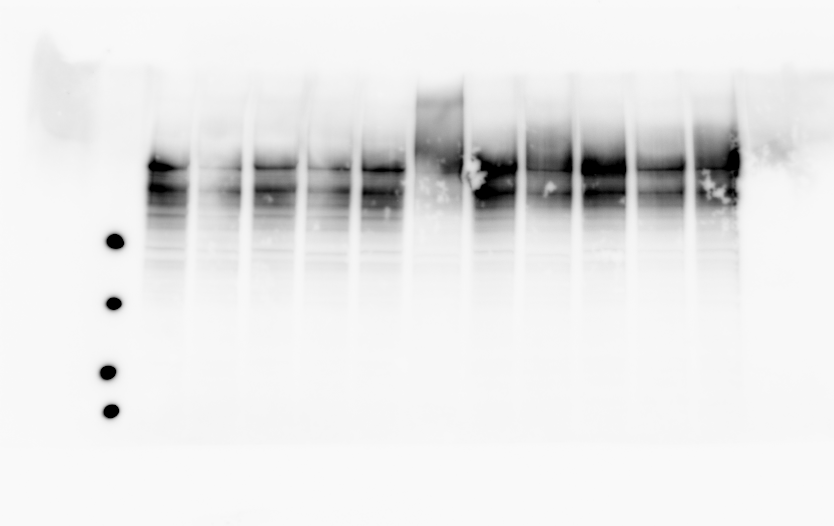

Supplement: Figure 6—source data 1. [file elife-77725-fig6-data1.zip › Source Data 1/Raw WB gel2 total RyR.tif]

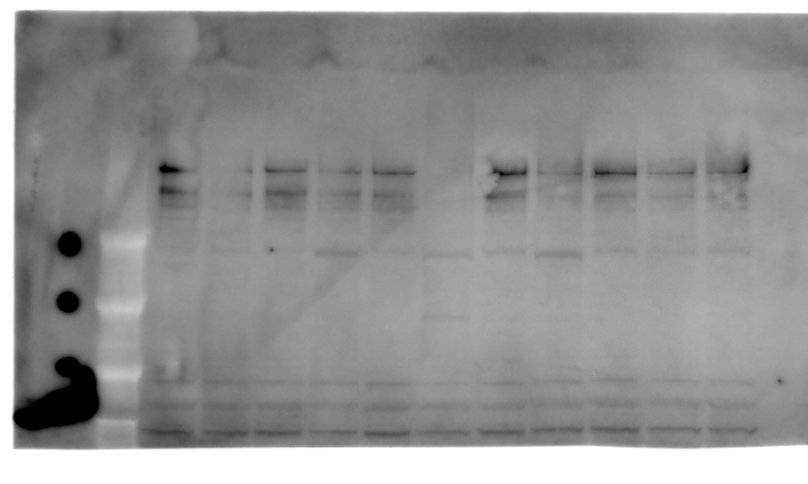

Supplement: Figure 6—source data 1. [file elife-77725-fig6-data1.zip › Source Data 1/Raw WB gel2a pser2808.tif]
