# Supplementary material for: Prolonged β-adrenergic stimulation disperses ryanodine receptor clusters in cardiomyocytes and has implications for heart failure
Source: eLife. 2022 Aug 1;11:e77725. doi: 10.7554/eLife.77725 (PMC9410709; doi:10.7554/eLife.77725)
Supplement: Supplementary file 1. — From a large number of automatically generated 3D Ca2+ release unit (CRU) geometries, four were selected to be used in mathematical modeling of Ca2+ release. These were chosen to represent the range of fragmentation observed experimentally, while ensuring that each CRU contained roughly the same number of RyRs. Characteristics for each CRU are listed. Spatial spread among the RyRs within each geometric configuration was summarized with calculation of the root mean square distance of RyRs to the CRU center. [file elife-77725-supp1.docx]

**Supplementary File 1**

|  | **σ (µm^2^/s)** | ***B*_tot_** | ***k*_on_ (μM^-1^.ms^-1^)** | ***k*_off_ (ms^-1^)** |
| --- | --- | --- | --- | --- |
| Calmodulin | 22 | 24 µM | 0.034 | 0.238 |
| ATP | 140 | 455 µM | 0.255 | 45 |
| Fluo | 20 | 50 µM | 0.08 | 0.09 |
| Troponin | 0 | 70 µM | 0.0327 | 0.0196 |
| Calsequestrin | 0 | 16 mM | 0.102 | 65 |
