# Supplementary material for: Prolonged β-adrenergic stimulation disperses ryanodine receptor clusters in cardiomyocytes and has implications for heart failure
Source: eLife. 2022 Aug 1;11:e77725. doi: 10.7554/eLife.77725 (PMC9410709; doi:10.7554/eLife.77725)
Supplement: Supplementary file 2. — Ca2+ diffusion is subject to mobile and immobile buffers in the cytosolic and sarcoplasmic reticulum (SR) compartments and was modeled for five buffer species (left) in kinetic detail. Only calsequestrin was modeled inside the SR domain, while the remaining buffers were cytosolic. Troponin and calsequestrin were modeled as immobile (σ = 0). The buffering capacity of each buffer species is defined by Btot, and the binding on- and off-rates are given by kon and koff, respectively. [file elife-77725-supp2.docx]

**Supplementary File 2**

|  | **σ (µm^2^/s)** | ***B*_tot_** | ***k*_on_ (μM^-1^.ms^-1^)** | ***k*_off_ (ms^-1^)** |
| --- | --- | --- | --- | --- |
| Calmodulin | 22 | 24 µM | 0.034 | 0.238 |
| ATP | 140 | 455 µM | 0.255 | 45 |
| Fluo | 20 | 50 µM | 0.08 | 0.09 |
| Troponin | 0 | 70 µM | 0.0327 | 0.0196 |
| Calsequestrin | 0 | 16 mM | 0.102 | 65 |
