# Supplementary material for: Prolonged β-adrenergic stimulation disperses ryanodine receptor clusters in cardiomyocytes and has implications for heart failure
Source: eLife. 2022 Aug 1;11:e77725. doi: 10.7554/eLife.77725 (PMC9410709; doi:10.7554/eLife.77725)
Supplement: Supplementary file 3. — Parameters of the two-state RyR model. The on-rate (+) and off-rate (-) for Ca2+ activation are given in the two rows of the table. For each rate, kmin and kmax represent the upper and lower bounds at very low and high cytosolic calcium ([Ca2+]i). These rate bounds apply when the corresponding value for ([Ca2+]i/Kd)n is outside of the bounds they set. Otherwise, both rates are calculated via the corresponding ([Ca2+]i/Kd)n. The on-rate was modified to simulate sensitization to cytosolic Ca2+ accompanying β-adrenergic receptor (β-AR) stimulation by shifting the half-maximal concentration from 45 µM to 25 µM, as denoted by *. [file elife-77725-supp3.docx]

**Supplementary File 3**

| **Rates** | *k*_min_ (ms^-1^) | *k*_max_ (ms^-1^) | K_d_ (µM) | n |
| --- | --- | --- | --- | --- |
| **+** | 5 x 10^-6^ | 0.9 | 45 or 25* | 2.7 |
| **-** | 0.9 | 3 | 45 | -0.5 |
| **Unitary Current** | 0.235 pA |  |  |  |
