# Supplementary material for: Prolonged β-adrenergic stimulation disperses ryanodine receptor clusters in cardiomyocytes and has implications for heart failure
Source: eLife. 2022 Aug 1;11:e77725. doi: 10.7554/eLife.77725 (PMC9410709; doi:10.7554/eLife.77725)
Supplement: Supplementary file 4. — HF development was examined by CINE MRI assessment based on established criteria; Sjaastad et al., 2000. Infarct size is percentage of left ventricular free wall (sham: nheart = 14; HF: nheart = 12; *p<0.05). [file elife-77725-supp4.docx]

**Supplementary File 4**

| **Parameter** | **Sham (n=14)** | **MI (n=12)** |
| --- | --- | --- |
| Infarct size (%) |  | 59.9 ± 2.1 |
| End-diastolic volume (mL) | 0.33 ± 0.01 | 0.03 ± 0.004* |
| End-systolic volume (mL) | 0.09 ± 0.01 | 0.02 ± 0.003* |
| Stroke volume (mL) | 0.24 ± 0.01 | 0.01 ± 0.001* |
| Ejection fraction | 0.73 ± 0.02 | 0.35 ±0.02* |
